# When the heart deceives: a case report of hyperthyroidism disguised as STEMI in female pregnant patient

**DOI:** 10.1186/s43044-025-00607-5

**Published:** 2025-01-15

**Authors:** Kevin Wibawa, Lidia Debby Wiyono, Raditya Dewangga, Arief Sumarna, Wizhar Syamsuri, Yandi Ariffudin, Suhendiwijaya Suhendiwijaya, Pangeran Akbar Syah

**Affiliations:** 1 Department of Cardiology and Vascular Medicine, Rumah Sakit Umum Daerah Gunung Jati, Kesambi Street No. 56, Cirebon, West Java 45134 Indonesia; 2 Department of Cardiology and Vascular Medicine, Rumah Sakit Hasan Sadikin, Bandung, Indonesia; 3 Department of Internal Medicine, Rumah Sakit Umum Daerah Gunung Jati, Kesambi Street No. 56, Cirebon, West Java 45134 Indonesia; 4 Faculty of Medicine, Universitas Swadaya Gunung Jati, Terusan Pemuda Street, Cirebon, West Java 45132 Indonesia

**Keywords:** Acute myocardial infarction, Hyperthyroidism, Pregnancy, STEMI

## Abstract

**Background:**

Acute myocardial infarction during pregnancy is a rare condition with an incidence of 1 to 10 per 100,000 deliveries. ST-elevation myocardial infarction (STEMI) is dominating the clinical presentation. It is estimated that 29% of the patients had normal coronary arteries, and hyperthyroidism may be associated with coronary vasospasm.

**Case Presentation**: A 30-year-old pregnant woman was referred with inferior STEMI post-failed fibrinolytic therapy. Her hospitalization course was complicated by non-sustained ventricular tachycardia and cardiogenic shock. Coronary angiography revealed normal coronary arteries without intracoronary thrombus, coronary dissection, or coronary atherosclerotic lesion. Laboratory test showed high Free T4 2.71 ng/dL and low TSH < 0.05 mlU/mL. Patient’s condition and hospitalization course were significantly improved after the initiation of hyperthyroid therapy. We suspected a hyperthyroid-induced coronary vasospasm as a potential etiology of myocardial infarction with non-obstructive coronary artery (MINOCA) in this patient.

**Conclusion:**

Although MINOCA due to hyperthyroidism is a rare finding among pregnant young woman, recognizing this etiology is a paramount of importance due to improved survival with appropriate and specific therapy.

**Supplementary Information:**

The online version contains supplementary material available at 10.1186/s43044-025-00607-5.

## Background

Acute myocardial infarction (AMI) during pregnancy is a rare condition but is associated with detrimental outcomes for both the mother and the fetus^1^. The incidence of AMI in pregnancy varied from 1 to 10 per 100,000 deliveries [2]. Among cases of AMI during pregnancy, 75% present as ST-elevation myocardial infarction (STEMI) and 25% as non-ST elevation myocardial infarction (NSTEMI) [3]. Intriguingly, coronary angiography revealed normal coronary arteries without atherosclerotic lesion or intracoronary thrombus in 29% of AMI during pregnancy. In patients with normal coronary artery, the underlying etiologies may consist of spontaneous coronary artery dissection (SCAD), coronary vasospasm, coronary embolism, and hyperthyroidism [3–5]. In young pregnant patients without significant classic cardiovascular risk factors, identifying other etiologies of AMI is of paramount importance and may improve patient outcomes. Here, we present the case of a 30-year-old pregnant woman with hyperthyroidism mimicking inferior STEMI.

## Case presentation

**Fig. 1:**
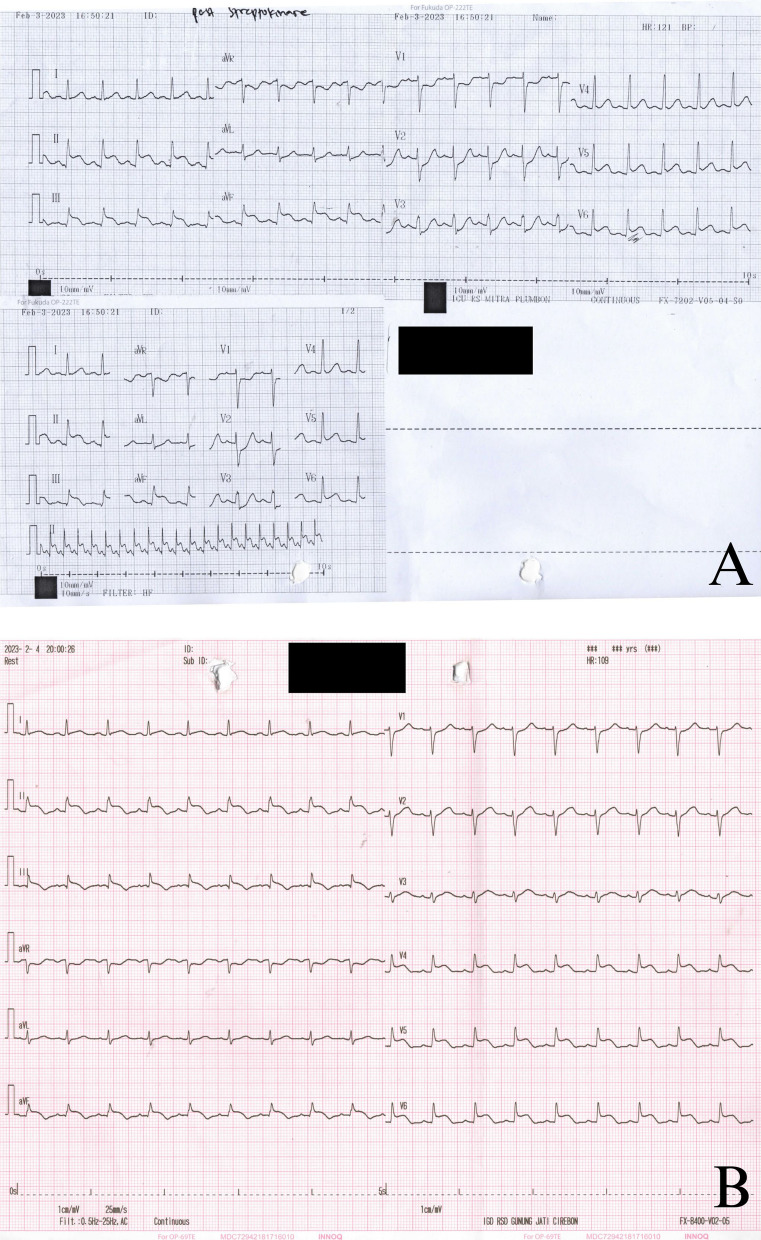
**A** Post-fibrinolytic ECG at the previous hospital; **B** ECG upon admission on our emergency department

A 30-year-old woman, gravida 3 para 2, at 8 weeks gestation, was referred to our hospital with an inferior and lateral ST-elevation myocardial infarction (STEMI). She denied any chest pain history or medical complications in the previous pregnancies. She had a spontaneous vaginal delivery in both of her previous obstetrical history. She was referred due to failed fibrinolytic therapy and persistent chest pain from other hospital (Fig. 1A). She reported experiencing an episode of crushing pain in the precordial area, accompanied by dyspnea and orthopnea, one day prior to hospitalization. She was treated with aspirin 320 mg, clopidogrel 300 mg, folic acid once daily (OD), atorvastatin 20 mg OD, and streptokinase 1.5 million UI in the previous hospital. Upon arrival on emergency department (ED,) the patient was hemodynamically stable.

Initial electrocardiogram (ECG) on our hospital revealed a persistent ST-segment elevation in inferior (II, III, aVF) and lateral (V4-V6) leads (Fig. 1B). Cardiac enzymes were significantly elevated. Kidney and liver function, complete blood count, electrolytes, and D-dimer were normal. Chest radiograph was not performed.

**Fig. 2:**
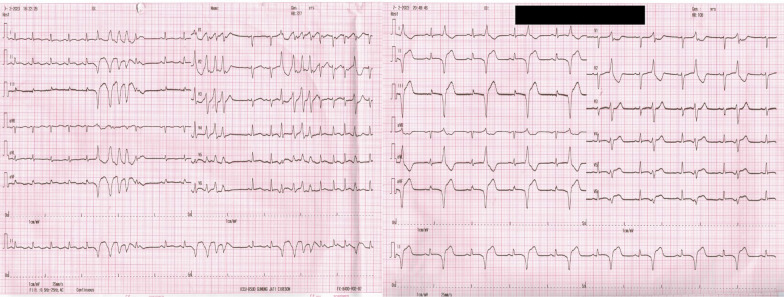
Non-sustained Ventricular Tachycardia and Bigeminal Premature Ventricular Complex

Within the first 3 h at the ED, she developed cardiogenic shock and was subsequently transferred to intensive cardiac care unit (ICCU) with dobutamine infusion 3 mcg/kg/min. She had an episode of non-sustained ventricular tachycardia (NSVT) and bigeminal premature ventricular complexes (PVCs) that responded to lidocaine (Fig. 2).

**Fig. 3:**
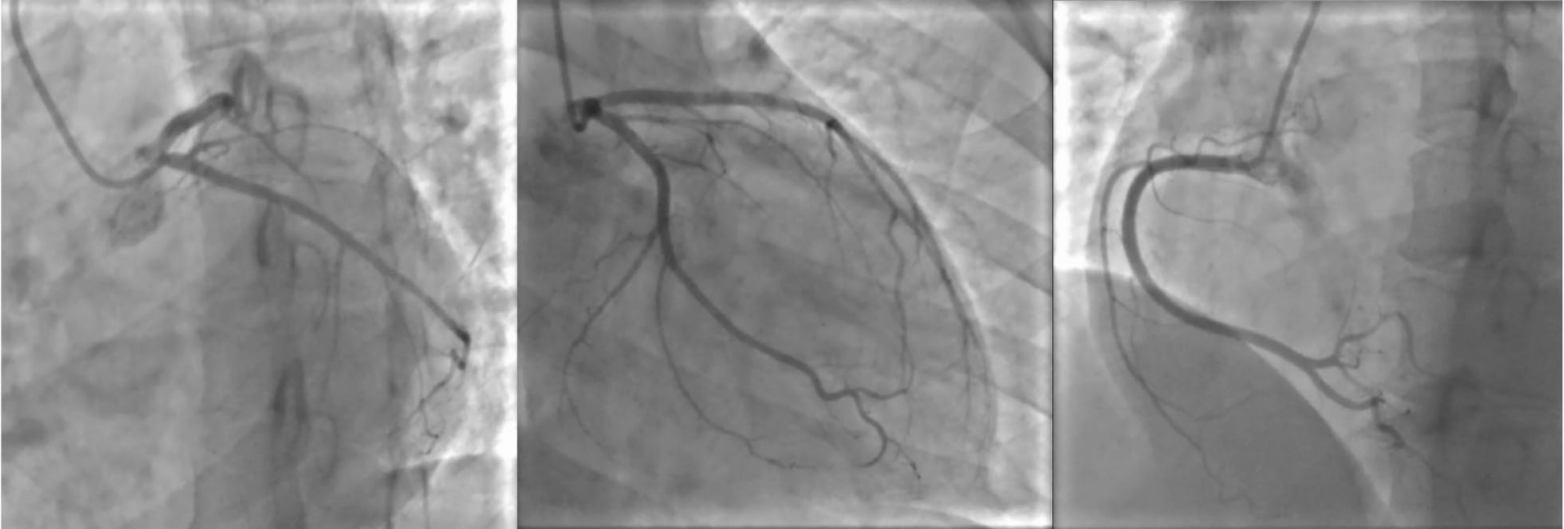
Patient’s coronary angiography

Emergent coronary angiography revealed normal coronary arteries (Fig. 3). Transthoracic echocardiography (TTE) showed left ventricular (LV) dilatation, reduced LV systolic function (LVEF 34.83% Teichholz), reduced right ventricular (RV) contractility, and global hypokinetic area; no valvular abnormalities are found (Supplementary Fig. 1).

Multidisciplinary approach was taken by involving cardiologist, internist, obstetrician, nurse and midwife. Fetal ultrasonography (USG) was performed daily after the patient had been stabilized. Fetal USG showed a single live intrauterine fetus at 13–14 weeks gestational age, with measurement of biparietal diameter, abdominal circumference, and fetus weight are in normal value. A blood sample was taken for additional electrolyte, procalcitonin, antinuclear antibody (ANA), thyroid function, and erythrocyte sedimentation rate (ESR) test. Her additional blood test showed hypokalemia 3.12 mmol/L (reference value 3.6–5.0 mmol/L), high level Free T4 2.71 ng/dL (reference value 0.82–1.50 ng/dL), low TSH < 0.05 mlU/mL (reference value 0.25–5.00 mlU/mL), high level ESR 1 38 (reference value < 20 mm), and high level ESR 2 80 (reference value < 20 mm). Procalcitonin and ANA Test were normal. A diagnosis of hyperthyroidism was established, and the patient was initiated on propylthiouracil 100 mg ter in die (TID), potassium chloride 600 mg TID, methylprednisolone 16 mg quarter in die (QD), and folic acid QD. The patient was discharged after 10 days of hospitalization with an intact fetal heart sounds. She was diagnosed with myocardial infarction with non-obstructive coronary artery (MINOCA) due to hyperthyroidism in pregnancy.

One month later, the patient was followed-up on cardiovascular outpatient clinic. Patient was asymptomatic and hemodynamically stable. TTE showed normal LV systolic function (LVEF 63.78%), good RV contractility, no RWMA, and mild mitral regurgitation (Supplementary Fig. 2). Indicating appropriate antithyroid therapy significantly improved the prognosis of patients with MINOCA due to hyperthyroidism. Upon follow-up on outpatient clinic, the thyroid parameters value were FT4 1.28 ng/dL and TSH 2.48 uIU/mL on May 08, 2024, and FT4 1.30 ng/d: and TSH 3.12 uIU/mL on August 16, 2024.

## Discussion

The incidence of acute myocardial infarction (AMI) during pregnancy is four times higher than in non-pregnant women of reproductive age [6]. Acute myocardial infarction related to pregnancy may have several etiologies. A significant proportion, up to 43% of cases, is attributed to spontaneous coronary artery dissection (SCAD). Recent literature suggests that coronary artery disease (CAD) is the second most prevalent cause. Uncommon etiologies include coronary thrombosis and coronary spasm in the absence of CAD [2, 7].

Coronary spasm is observed in 2% of acute myocardial infarction related to pregnancy cases, and hyperthyroidism has been linked with coronary artery spasm [5]. Hyperthyroidism may lead to a hyperkinetic circulatory system with tachycardia, an increase in pulse pressure, and a decrease in peripheral resistance as a result of vasorelaxation [8, 9]. An elevated metabolic state caused by hyperthyroidism triggers an imbalance between blood supply and oxygen demand which further can cause MINOCA (myocardial infarction with non-obstructive coronary arteries) [2].

Although no direct evidence of coronary vasospasm was observed during coronary angiography, we speculated that vasospasm was a potential etiology in this patient. Coronary angiography showed no dissection, thrombus, or atherosclerosis. However, persistent ST-segment elevation and elevated troponin levels indicated myocardial damage, which could not be explained by other causes.

Based on findings of this patient, the diagnosis of MINOCA due to hyperthyroidism was established. MINOCA is an increasingly recognized entity with a variety of potential underlying mechanisms. The diagnosis of MINOCA requires: (i) clinical documentation of myocardial infarction, (ii) exclusion of obstructive coronary arteries, and (iii) no overt cause for presenting with AMI, such as cardiac trauma [10]. Precordial chest discomfort and ST-segment elevation myocardial infarction were detected in this patient; however, coronary angiography revealed normal coronary arteries with no other causes [10]. Cardiac magnetic resonance imaging (CMRI) should be the initial diagnostic test for the evaluation of the cardiac causes of MINOCA. In patients with MINOCA, a provocative spasm test may also be considered [10]. Unfortunately, CMRI and provocative spasm testing were not performed on our patient due to limited resources.

The standard of care for a pregnant woman with MI should be the same as the standard of care for a non-pregnant woman with the necessary changes [5], although the evidences were limited [11]. Identification of treatable causes of MINOCA is fundamental to their clinical evaluation, as it can affect their prognosis and prognostic value [12].

## Conclusion

MINOCA due to hyperthyroidism is a rare finding among pregnant young woman. This etiology should be suspected in every pregnant young woman without significant comorbidities who presented with STEMI on ECG and normal coronary angiogram. Despite eventful hospitalization course, MINOCA due to hyperthyroidism is a potentially curable and appropriate treatment significantly improved patient’s condition.

## Supplementary Information

Additional file 1.

## Data Availability

The datasets used and/or analyzed during the current study are available from the corresponding author upon on reasonable request.

## Abbreviations

AMI: Acute myocardial infarction
CAD: Coronary artery disease
CMRI: Cardiac magnetic resonance imaging
ECG: Electrocardiography
ED: Emergency department
ICCU: Intensive cardiovascular care unit
LVEF: Left-ventricular ejection fraction
LV: Left ventricle
MINOCA: Myocardial infarction with non-obstructive coronary artery
NSTEMI: Non-ST segment myocardial infarction
NSVT: Non-sustained ventricular tachycardia
OD: Once daily
PVC: Premature ventricular complex
QID: Quarter in die
RWMA: Regional wall motion abnormality
RV: Right ventricle
SCAD: Spontaneous coronary artery dissection
STEMI: ST-elevation myocardial infarction
TID: Ter in die
TTE: Tranthoracic echocardiography
USG: Ultrasonography

## Footnotes

1 From one study, the maternal mortality rate may reach as high as 11% in women of reproductive age [1]
