# Supplementary material for: When the heart deceives: a case report of hyperthyroidism disguised as STEMI in female pregnant patient
Source: Egypt Heart J. 2025 Jan 15;77:12. doi: 10.1186/s43044-025-00607-5 (PMC11735702; doi:10.1186/s43044-025-00607-5)
Supplement: Supplementary file 1 — Additional file 1. [file 43044_2025_607_MOESM1_ESM.docx]

Supplementary Data


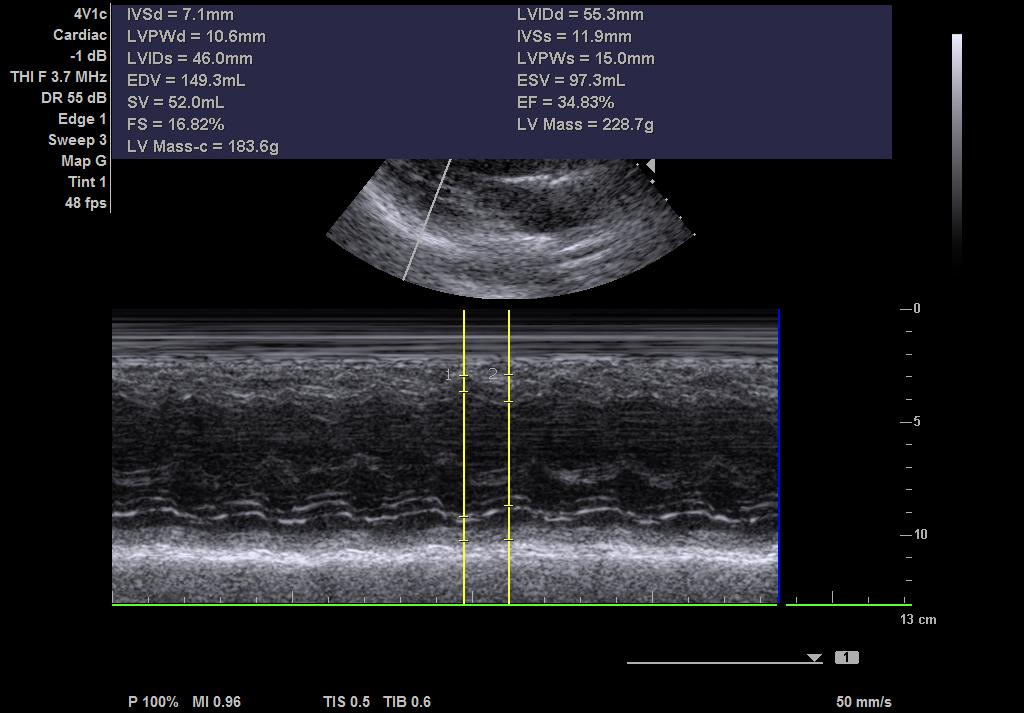


Supplementary Figure 1. Baseline left-ventricular ejection fraction during hospitalization


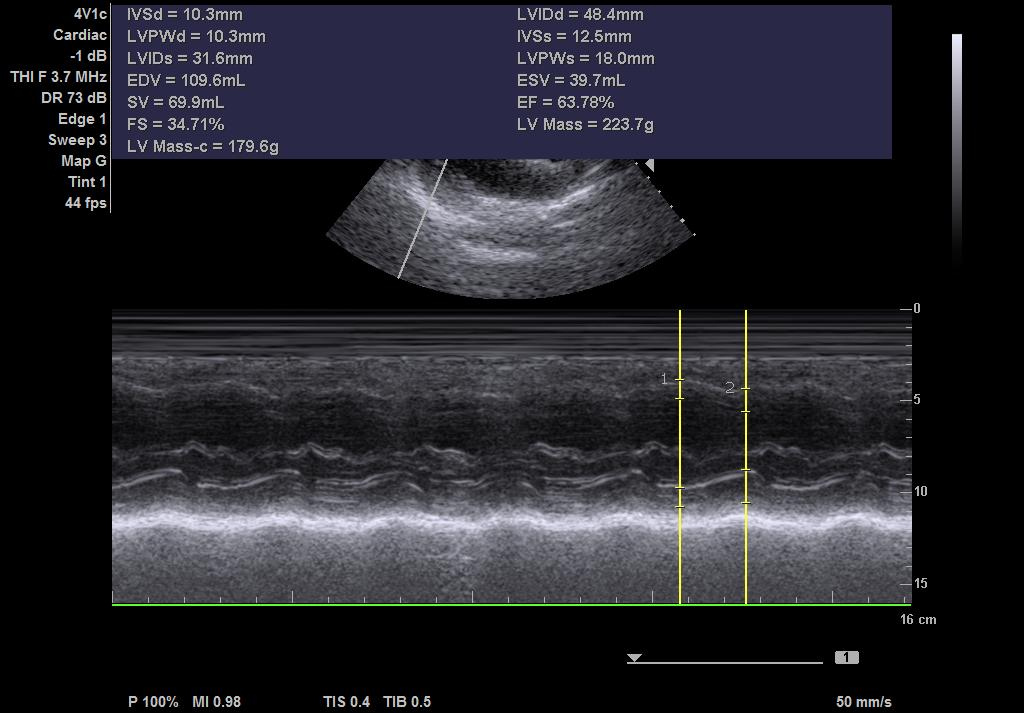


Supplementary Figure 2. Left-ventricular ejection fraction on follow-up on outpatient clinic
